# The Relationship Between Physical Activity Level, Respiratory Muscle Strength, and Cough Strength in Individuals with Type 2 Diabetes Mellitus: An Observational Cross-Sectional Study

**DOI:** 10.3390/jcm15124566

**Published:** 2026-06-12

**Authors:** Ayşenur Yılmaz, Halil Yılmaz, Nilufer Cetisli-Korkmaz, Semin Melahat Fenkçi, Goksel Altinisik, Betül Söylemez, Esra Yılmaz Bektaş

**Affiliations:** 1 Department of Physiotherapy and Rehabilitation, Faculty of Health Sciences, Burdur Mehmet Akif Ersoy University, 15030 Burdur, Türkiye; 2 Department of Internal Medicine, Faculty of Medicine, Pamukkale University, 20160 Denizli, Türkiye; halilyilmaz@pau.edu.tr (H.Y.); fenkci@yahoo.com (S.M.F.); 3 Faculty of Physiotherapy and Rehabilitation, Pamukkale University, 20160 Denizli, Türkiye; niluferc75@yahoo.com (N.C.-K.); ylmzesra@hotmail.com (E.Y.B.); 4 Department of Chest Diseases, Faculty of Medicine, Pamukkale University, 20160 Denizli, Türkiye; gkiter@pau.edu.tr; 5 Department of Medical Services and Techniques, Burdur Vocational School of Health Services, Burdur Mehmet Akif Ersoy University, 15030 Burdur, Türkiye; bsoylemez@mehmetakif.edu.tr

**Keywords:** type 2 diabetes mellitus, physical activity level, respiratory muscle strength, cough

## Abstract

**Background/Objectives:** Pulmonary complications and respiratory muscle weakness are common in individuals with type 2 diabetes mellitus (Type 2 DM). Physical inactivity may further contribute to impaired respiratory muscle performance and reduced cough effectiveness in this population. The aim of this study was to examine the relationship between physical activity level, respiratory muscle strength, and cough strength in individuals with Type 2 DM. This study was an observational cross-sectional study. **Methods:** Thirty individuals with Type 2 DM and thirty age- and sex-matched healthy controls were included. Their physical activity level was assessed using the International Physical Activity Questionnaire (IPAQ), and respiratory muscle strength was evaluated using maximal inspiratory pressure (MIP) and maximal expiratory pressure (MEP), whereas cough strength was assessed using a peak expiratory flow meter. Group comparisons were performed using the Mann–Whitney U test, and associations were analyzed using Spearman’s correlation coefficients. Additional multivariable regression analyses were conducted after adjustment for body mass index (BMI) and waist circumference. **Results:** Physical activity level, respiratory muscle strength, and cough strength were significantly lower in individuals with Type 2 DM than in healthy controls (*p* < 0.05). Within the Type 2 DM group, IPAQ scores were positively correlated with MIP (r = 0.438, *p* = 0.016), MEP (r = 0.581, *p* = 0.001), and cough strength (r = 0.619, *p* < 0.001), and diabetes duration was negatively correlated with MIP (r = −0.412, *p* = 0.024). After adjustment for BMI and waist circumference, Type 2 DM status remained independently associated with lower MIP (B = −15.69, 95% CI: −26.58 to −4.80, *p* = 0.006) and lower cough strength (B = −90.51, 95% CI: −161.52 to −19.50, *p* = 0.013). **Conclusions:** Individuals with Type 2 DM demonstrated lower physical activity levels, respiratory muscle strength, and cough strength than healthy controls. Higher physical activity levels were associated with better respiratory muscle strength and cough strength, whereas longer diabetes duration was associated with lower inspiratory muscle strength. These findings suggest that respiratory muscle function and cough performance may be adversely affected in individuals with Type 2 DM, warranting further investigation in larger longitudinal studies.

## 1. Introduction

Type 2 diabetes mellitus (type 2 DM) is one of the most common chronic metabolic diseases worldwide and represents a major global public health problem due to its rapidly increasing prevalence and associated complications [1]. According to the International Diabetes Federation (IDF) Diabetes Atlas 2025, approximately 589 million adults worldwide are living with diabetes, and this number is projected to increase to 853 million by 2050 [2]. Chronic complications negatively affect respiratory function in individuals with type 2 DM. Type 2 DM causes changes in body composition, increased abdominal fat, reduced cardiopulmonary fitness, and reduced quality of life due to microvascular and macrovascular complications [3].

Microangiopathy and non-enzymatic glycosylation of tissue proteins contribute to the development of chronic complications in DM. The lungs, which are rich in connective tissue and contain an extensive microvascular network, may also be affected in DM [4]. Hyperglycemia can directly damage lung tissue through glycosylation (via advanced glycation end products) and inflammation mediated through circulation [1]. Autonomic neuropathy due to microangiopathy is observed in patients with DM, and diabetic neuropathy contributes to deterioration in respiratory function [5]. In type 2 DM, respiratory dysfunction and respiratory muscle weakness may result in impaired lung function, ineffective cough, and lung complications [6]. Additionally, decreased respiratory muscle strength can affect both respiratory and nonrespiratory functions such as coughing, speaking, and swallowing. Cough augmentation helps clear the airways to reduce the risk of respiratory tract infections. Increasing lung volume prevents or reverses atelectasis and chest-wall stiffening [7]. Cough is an important defense mechanism that is directly related to respiratory muscle strength and depends on the ability to generate airflow and velocity within the airways [8]. Several types of lung infections, including pneumonia, tuberculosis, and atelectasis, have been reported to occur more frequently in patients with type 2 DM over time [9]. Cough strength assessment using peak cough flow has been widely utilized in neuromuscular diseases and critically ill patients as a practical and reliable method for evaluating airway clearance capacity and predicting respiratory outcomes [8,10,11]. An effective cough is essential for airway protection and the prevention of respiratory complications. Individuals with type 2 DM are known to have an increased risk of respiratory muscle weakness and pulmonary complications due to chronic hyperglycemia, systemic inflammation, and diabetic neuropathy [5,9]. Despite these respiratory impairments, cough strength has not been sufficiently investigated in individuals with type 2 DM. Evaluating cough strength in this population may provide important clinical information regarding airway defense mechanisms and potential respiratory complications. One study reported that cough strength is among the most reliable methods for evaluating cough severity, particularly in patients with neuromuscular disorders, and is commonly assessed using peak expiratory flow meters [10]. It has also been reported to be a good predictor of extubation outcomes and hospital mortality in patients without neuromuscular disease [11]. Assessment of cough strength using a peak expiratory flow meter may provide a simple, inexpensive, and clinically practical method for evaluating airway defense capacity in individuals with type 2 DM. Beyond its role in critically ill and mechanically ventilated patients, cough strength may also serve as a functional indicator of respiratory muscle performance in individuals with chronic diseases. Because effective cough depends on adequate inspiratory and expiratory muscle function, impairments in respiratory muscle strength may be reflected in reduced cough effectiveness. Peak cough flow assessment is simple, rapid, non-invasive, and readily applicable in routine clinical practice. Therefore, establishing the relationship between cough strength and respiratory muscle strength may help clinicians identify early respiratory dysfunction using a practical functional assessment tool in individuals with type 2 DM. However, cough strength assessment has been understudied in individuals with type 2 DM.

Physical activity is an important determinant of respiratory health in individuals with chronic diseases. Reduced physical activity levels are frequently observed in individuals with type 2 DM due to obesity, fatigue, decreased exercise tolerance, and diabetes-related complications [12,13]. In addition, reduced physical activity has been associated with sarcopenia, impaired physical performance, and functional decline in this population [14]. Previous studies have shown that regular physical activity positively affects pulmonary function, aerobic capacity, and overall functional status, and physically active individuals tend to demonstrate better respiratory muscle performance and respiratory efficiency [15]. Furthermore, a recent systematic review highlighted that obesity and type 2 DM may adversely affect lung health and respiratory function [16]. Importantly, inspiratory muscle training has been reported to improve respiratory muscle performance and exercise-related outcomes in individuals with type 2 DM, suggesting that respiratory dysfunction may be modifiable through targeted interventions [17]. Because effective coughing depends on adequate respiratory muscle strength, physical activity may indirectly contribute to airway protection and cough effectiveness by improving respiratory muscle performance. Assessment of cough strength is particularly useful because it provides a simple, practical, and inexpensive method for evaluating airway defense mechanisms. Furthermore, the relationship between physical activity level and cough strength in this population remains insufficiently explored. To the best of our knowledge, no previous study has simultaneously evaluated physical activity level, respiratory muscle strength, and cough strength in individuals with type 2 DM or examined the relationships among these variables. This knowledge gap provided the rationale for the present study.

Although previous studies have separately examined pulmonary function, respiratory muscle strength, physical activity, and respiratory muscle training in individuals with type 2 DM, the relationship between physical activity level, respiratory muscle strength, and cough strength has not been sufficiently investigated in this population. Furthermore, whether cough strength could provide a practical functional indicator of respiratory muscle impairment in individuals with type 2 DM remains unclear.

This study aimed to examine the relationship between physical activity level, respiratory muscle strength, and cough strength in individuals with type 2 DM. Based on the adverse effects of physical inactivity on respiratory muscle performance and pulmonary function, we hypothesized that lower physical activity levels would be associated with reduced respiratory muscle strength and decreased cough strength in individuals with type 2 DM.

## 2. Materials and Methods

### 2.1. Study Design

This observational cross-sectional study was conducted at the Pamukkale University Department of Internal Medicine, Endocrine and Metabolic Diseases Polyclinic, and the Faculty of Physiotherapy and Rehabilitation. All procedures were conducted in accordance with the ethical standards of the Declaration of Helsinki. This study was approved by the Pamukkale University Non-Interventional Clinical Research Ethics Committee (60116787-020/5391). Participant recruitment and data collection were conducted over an extended period within the scope of a TÜBİTAK-supported project. In addition, data analysis, manuscript preparation, journal submission, and revision contributed to the time interval between ethics approval and manuscript submission.

### 2.2. Participants

**Figure 1:**
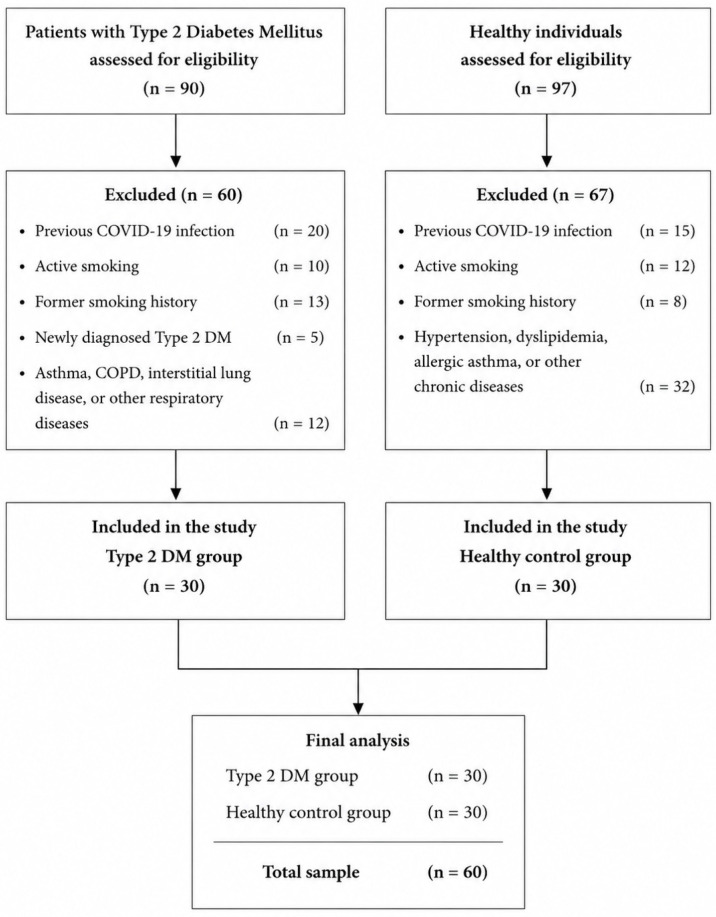
Participant recruitment, eligibility assessment, exclusions, and final study sample.

Participants were recruited using a convenience sampling method from individuals who met the study eligibility criteria and volunteered to participate. Individuals aged ≥19 years who had been diagnosed with type 2 DM according to the American Diabetes Association criteria [18], had been using oral antidiabetic drugs for at least 1 year, and had no history of respiratory diseases such as asthma, chronic obstructive pulmonary disease, or interstitial lung disease were enrolled in the type 2 DM group. Individuals with a current or previous smoking history and those with a previous history of COVID-19 were excluded. Age- and sex-matched healthy individuals with HbA1c levels between 4% and 6%, no respiratory symptoms or respiratory disease such as asthma, chronic obstructive pulmonary disease, or interstitial lung disease, no smoking history, and no previous history of COVID-19 were included in the control group. All participants included in the final analysis completed all study assessments. Individuals who were unable to complete the assessments were excluded from the study. A flowchart illustrating participant recruitment, eligibility assessment, exclusions, and final group allocation is presented in Figure 1.

### 2.3. Sample Size

The reference study reported a large effect size (d = 0.78). To provide a more conservative estimate, the sample size calculation was based on an effect size of d = 0.7. Power analysis indicated that a minimum of 52 participants (26 per group) would provide 80% power at a 95% confidence level [19].

### 2.4. Outcome Measurements

In addition to the participants’ descriptive characteristics, their occupation, alcohol use, and exercise habits were recorded using a demographic data form. After determining whether participants had experienced a respiratory tract infection within the previous year, routine blood results obtained during the preceding 3 months [fasting glucose, high-density lipoprotein (HDL), low-density lipoprotein (LDL), total cholesterol, triglycerides, HbA1C, and vitamin B12 levels] were retrieved from the hospital electronic database. All respiratory muscle strength and cough strength assessments were performed by the same experienced physiotherapist, ensuring consistency in measurement procedures; therefore, inter-rater reliability was not applicable. All assessments were performed in the same sequence, with appropriate rest periods between tests. To ensure standardization, evaluations were conducted during similar afternoon hours in both groups. Due to the observational study design, assessor blinding to group allocation was not feasible. Before testing, all participants received standardized verbal instructions and demonstrations. At least three technically acceptable maneuvers were performed for each measurement, and the highest value was recorded.

Respiratory muscle strength was evaluated using a Cosmed Pony FX^®^ (COSMED Srl, Rome, Italy) device. Maximum inspiratory pressure (MIP) and maximum expiratory pressure (MEP) were measured. The highest maximal pressure value (cmH_2_O) obtained from at least 3 acceptable attempts was recorded [20].

Cough strength was evaluated using a PEF meter (AirZone Peak flow meter^®^ (Clement Clarke International Ltd., Harlow, UK)). Participants were asked to cough as forcefully as possible after a deep inspiration (maximum waiting time: 2 s). The cough strength generated during the maneuver was recorded. The test was repeated three times, and the highest value was recorded in L/min [8,21]. Peak cough flow measurements were evaluated together with reference expiratory flow values derived from the regression equations developed by Nunn and Gregg [22]. Although these equations were originally developed for peak expiratory flow assessment, they were used in the present study to provide reference expiratory flow values for comparison purposes, given the physiological relationship between peak expiratory flow and cough flow generation. In addition, peak cough flow assessment was supported by current literature on cough flow evaluation [8].

Physical activity level was determined using the short-form version of the International Physical Activity Questionnaire (IPAQ). According to the IPAQ, individuals’ activity levels are expressed in metabolic equivalents (METs) [23].

### 2.5. Statistical Analysis

All calculations were performed using the Statistical Package for the Social Sciences (IBM SPSS Statistics for Windows, Version 21.0 (IBM Corp., Armonk, NY, USA)). The normality of the data distribution was assessed using the Shapiro–Wilk test. Since most variables did not meet the assumptions of normality, continuous variables were presented as median (25th–75th percentile), whereas categorical variables were expressed as frequencies and percentages. Differences between independent groups were analyzed using the Mann–Whitney U test. Relationships between continuous variables were evaluated using Spearman’s correlation coefficient. Correlation strength was classified as follows: 0.00, no relationship; 0.01–0.29, low; 0.30–0.70, moderate; 0.71–0.99, high; and 1.00, excellent [24]. A *p* value < 0.05 was considered statistically significant.

To evaluate whether type 2 DM status was independently associated with respiratory outcomes, additional multivariable linear regression analyses were performed. Respiratory muscle strength (MIP and MEP) and cough strength were used as dependent variables, whereas group status (type 2 DM vs. healthy controls) was entered as the independent variable. BMI and waist circumference were included as covariates because they differed significantly between groups and may influence respiratory performance. Unstandardized regression coefficients (B), 95% confidence intervals (CI), and *p* values were reported.

## 3. Results

Thirty patients with type 2 DM and thirty healthy controls individually matched for age and sex were included in the study, and all participants completed the planned assessments. There were nineteen (63.3%) female participants in each group. Although there was no statistically significant difference in age or height between the groups (*p* > 0.05), BMI, body weight, and the waist-to-hip ratio were significantly higher in individuals with type 2 DM (*p* < 0.05, Table 1). The mean disease duration was 7.73 ± 3.81 years; 11 participants (36.6%) had a disease duration of 1–5 years, 8 (26.4%) had a disease duration of 5–10 years, and 11 (36.6%) had a disease duration of more than 10 years.

Among the 12 participants with diabetes-related complications, 2 (16.7%) had isolated neuropathy, 4 (33.3%) had both retinopathy and neuropathy, 5 (41.7%) had both nephropathy and neuropathy, and 1 (8.3%) had nephropathy, neuropathy, and retinopathy. The fasting blood glucose, postprandial blood glucose, triglyceride, and HbA1c values in the type 2 DM group were significantly higher than those in the healthy control group (*p* < 0.05). Although median LDL levels were higher in the healthy control group (*p* < 0.01), the groups were similar with respect to median HDL, total cholesterol, vitamin B12, and folic acid levels. Systolic and diastolic blood pressure values were significantly higher in individuals with Type 2 DM than in healthy controls (*p* < 0.05, Table 1).

According to the IPAQ classification, only 5 participants (16.7%) in the Type 2 DM group were categorized as highly active, 3 (10.0%) as minimally active, and 22 (73.3%) as inactive. In contrast, among the healthy controls, 15 participants (50.0%) were highly active, 9 (30.0%) were minimally active, and 6 (20.0%) were inactive (*p* < 0.05). Consistent with these findings, the median IPAQ scores were significantly lower in individuals with Type 2 DM than in the healthy controls. In addition, cough strength, respiratory muscle strength (MIP and MEP), and their predicted percentage values were significantly lower in individuals with Type 2 DM than in healthy controls (*p* < 0.05, Table 2).

Within the Type 2 DM group, IPAQ scores showed moderate positive correlations with respiratory muscle strength (maximum inspiratory pressure [MIP] and maximum expiratory pressure [MEP]) and cough strength (r = 0.438, *p* = 0.016; r = 0.581, *p* = 0.001; and r = 0.619, *p* < 0.001, respectively). A negative correlation trend was observed between HbA1c levels and IPAQ scores; however, this relationship did not reach statistical significance (r = −0.352, *p* = 0.056). In the healthy control group, the IPAQ scores were positively correlated with cough strength (r = 0.470, *p* = 0.010) and MEP (r = 0.466, *p* = 0.011), whereas no significant correlations were observed between the IPAQ scores and MIP or HbA1c levels (Table 3).

Additional correlation analyses in the Type 2 DM group demonstrated that diabetes duration was negatively correlated with MIP (r = −0.412, *p* = 0.024). Furthermore, HbA1c levels were negatively correlated with MIP (r = −0.369, *p* = 0.045). No significant correlations were observed between BMI or waist-to-hip ratio and respiratory outcomes (Table 4).

Additional multivariable regression analyses adjusted for BMI and waist circumference demonstrated that Type 2 DM status remained independently associated with lower MIP (B = −15.69, 95% CI: −26.58 to −4.80, *p* = 0.006) and lower cough strength (B = −90.51, 95% CI: −161.52 to −19.50, *p* = 0.013). However, the association between Type 2 DM status and MEP did not remain statistically significant after adjustment (B = −14.29, 95% CI: −29.05 to 0.46, *p* = 0.057) (Table 5).

## 4. Discussion

We found that physical activity level, respiratory muscle strength, and cough strength were lower in individuals with type 2 DM than in age- and sex-matched healthy controls. In addition, respiratory muscle strength and cough strength were associated with physical activity level. To the best of our knowledge, this is one of the first studies to simultaneously evaluate physical activity level, respiratory muscle strength, and cough strength in individuals with Type 2 DM.

Adopting and maintaining physical activity are critical for blood sugar management and overall health in individuals with DM and prediabetes. Exercise is widely accepted as an important component of DM treatment. In individuals with DM, exercise provides cardiovascular benefits, aids weight management, and improves glycemic control, thereby reducing cardiovascular risk and mortality. To the best of our knowledge, no previous study has comprehensively evaluated the relationship between physical activity, respiratory muscle strength, and cough strength in individuals with type 2 DM. Previous studies have examined the relationship between physical activity and pulmonary function in older adults and healthy individuals [19,25,26,27]. Other studies have reported associations between physical activity level and respiratory muscle strength [19]. Respiratory muscle function, particularly the maximum inspiratory pressure (MIP), has been reported to be significantly associated with obesity indices (BMI) and body composition parameters (fat % and muscle mass %) at moderate and high physical activity levels [27].

Physical inactivity is closely associated with obesity, hypertension, smoking, and cholesterol levels. It contributes directly to the development of chronic cardiovascular disease and metabolic syndrome. In addition, physical inactivity in individuals with DM contributes to disease progression and increases morbidity and mortality [28]. In the present study, IPAQ scores were significantly lower in individuals with type 2 DM than in healthy controls. We also found moderate positive associations between cough strength, respiratory muscle strength, and physical activity level. Our findings are supported by the recent systematic review by Breuil-Marsal et al. [17], which reported that inspiratory muscle training may improve respiratory muscle performance and exercise-related outcomes in individuals with Type 2 DM. Although inspiratory muscle training differs from habitual physical activity, both findings highlight the potential importance of maintaining respiratory muscle function in this population. It should be noted that low physical activity, which is closely related to obesity, may adversely affect respiratory muscle strength and cough strength. More importantly, abdominal obesity is directly associated with respiratory problems because the accumulation of adipose tissue in and around the abdominal region mechanically affects the diaphragm and indirectly reduces lung volume, cough strength, and chest wall compliance [29]. Increasing physical activity, one of the most important strategies for combating obesity, may help improve muscle strength through both biomechanical and physiological mechanisms. Therefore, higher physical activity levels may be associated with better preservation of respiratory muscle strength and cough performance in individuals with Type 2 DM.

Interestingly, positive associations between physical activity level and both cough strength and expiratory muscle strength were also observed in healthy controls. These findings support the idea that regular physical activity may contribute to better respiratory muscle performance not only in individuals with Type 2 DM but also in otherwise healthy adults. However, the stronger and more consistent associations observed in the Type 2 DM group may suggest greater susceptibility of respiratory function to physical inactivity in this population.

In the Type 2 DM group, BMI was not significantly associated with respiratory muscle strength or cough strength, whereas physical activity level demonstrated significant positive correlations with MIP, MEP, and cough strength. These findings suggest that the observed impairments in respiratory muscle strength and cough performance cannot be explained solely by differences in body composition or abdominal obesity. Although obesity may negatively affect respiratory mechanics and respiratory performance, it may not fully explain the observed reductions in respiratory muscle strength and cough effectiveness. Respiratory muscle dysfunction may occur even in the absence of marked abnormalities in conventional pulmonary function parameters, indicating that respiratory muscle performance represents a distinct component of respiratory health. Given the multifactorial nature of Type 2 DM, including chronic hyperglycemia, diabetic neuropathy, systemic inflammation, reduced physical activity, and metabolic alterations, respiratory impairment is likely influenced by multiple interacting mechanisms rather than obesity alone. The relatively lower LDL cholesterol levels observed in the Type 2 DM group may be related to the use of lipid-lowering medications, particularly statins, which are commonly prescribed in individuals with type 2 diabetes for cardiovascular risk reduction. Furthermore, individuals with Type 2 DM are known to be more susceptible to respiratory complications and infections [9,30]. Therefore, practical and easily applicable methods for the early identification of respiratory dysfunction may be clinically valuable in this population.

An important finding of the present study was that the associations between Type 2 DM and both inspiratory muscle strength and cough strength remained significant after adjustment for BMI and waist circumference. This interpretation is consistent with recent evidence suggesting that Type 2 DM may adversely affect respiratory health through mechanisms beyond obesity alone, including chronic hyperglycemia, systemic inflammation, and microvascular alterations affecting pulmonary and respiratory muscle function [16].

These findings suggest that the observed impairments in inspiratory muscle strength and cough performance cannot be explained solely by obesity-related factors or abdominal adiposity and may reflect diabetes-specific mechanisms affecting respiratory muscle function.

Furthermore, BMI was not significantly correlated with respiratory muscle strength or cough strength within the Type 2 DM group. Other diabetes-related mechanisms, including chronic hyperglycemia, diabetic neuropathy, systemic inflammation, reduced physical activity, and metabolic alterations, may contribute to respiratory muscle dysfunction in individuals with Type 2 DM. Additionally, longer diabetes duration was associated with lower inspiratory muscle strength, suggesting that cumulative metabolic burden may progressively impair respiratory muscle performance over time.

Cough strength assessment is a useful tool for evaluating the risk of pulmonary complications and predicting extubation outcomes [30,31]. Because cough strength is sensitive to pulmonary complications, its assessment may also be clinically useful in individuals with muscle weakness. In a study investigating the effects of a four-week expiratory muscle strength training program on cough reflex parameters in inactive older adults, expiratory muscle training was reported to increase MEP and PEF values. Expiratory muscle strength is closely related to cough strength [31].

For an effective cough, peak cough flow should be above 160 L/min [32]. The cough reflex may become inadequate due to weakness of the upper respiratory tract and inspiratory and expiratory muscles. Ineffective coughing may lead to respiratory failure and aspiration pneumonia. An effective cough reflex is an important defense mechanism for preventing aspiration and facilitating secretion clearance. Increased airway resistance may reduce pulmonary volumes and contribute to impaired respiratory function. Lung infections, including pneumonia and tuberculosis, have been reported to be more common in individuals with type 2 DM because increased airflow resistance and alveolar damage may contribute to deterioration in lung function [33]. In the present study, although the median cough strength value in the Type 2 DM group remained above the threshold generally considered necessary for effective cough function, it was significantly lower than that in healthy controls. Nevertheless, this significant reduction compared with healthy controls may indicate a decline in airway clearance capacity before clinically overt respiratory dysfunction becomes apparent. These findings may indicate early impairment of airway defense mechanisms in individuals with type 2 DM. Collectively, these findings suggest that respiratory muscle performance and cough strength may represent important components of respiratory health assessment in individuals with Type 2 DM.

Individuals with a history of COVID-19 or respiratory disease were excluded to minimize the direct effects of these conditions on respiratory function and cough performance. However, individuals with type 2 DM are clinically more vulnerable to respiratory complications, and respiratory impairment may still develop even in the absence of overt respiratory disease. Therefore, the lower respiratory muscle strength and cough strength values observed in the present study may reflect early or subclinical respiratory involvement associated with type 2 DM.

### Limitations

This study has several limitations. First, due to the observational cross-sectional design, causal relationships between physical activity level, respiratory muscle strength, and cough strength cannot be established. Second, physical activity level was assessed using the self-reported IPAQ short form, which may be subject to recall and social desirability bias. Third, although individuals with Type 2 DM and healthy controls were matched for age and sex, significant differences in BMI, waist circumference, and blood pressure were observed between groups. Although additional multivariable regression analyses adjusting for BMI and waist circumference were performed, residual confounding by other clinical and metabolic factors cannot be excluded. Fourth, the study was conducted at a single institution in Turkey, which may limit the generalizability of the findings to other populations and settings. In addition, assessor blinding was not feasible because of the observational nature of the study, which may have introduced measurement bias. Another limitation is the absence of comprehensive pulmonary function testing, which may have provided additional insight into the mechanisms underlying respiratory impairment. In addition, the healthy control group was recruited on a voluntary basis. Therefore, the relatively high proportion of physically active individuals in this group may not fully reflect the physical activity distribution of the general population, which may limit the generalizability of the findings. Furthermore, subgroup analyses according to physical activity level within the Type 2 DM group were not performed because of the limited sample size. Future studies with larger samples should compare physically active and inactive individuals with Type 2 DM separately to better clarify the independent contribution of physical activity to respiratory outcomes. Finally, only individuals with Type 2 DM who had been using oral antidiabetic drugs for at least one year were included, and individuals with respiratory disease, a smoking history, and previous COVID-19 infection were excluded to minimize factors that directly affect respiratory function. Therefore, the findings may not fully represent all individuals with Type 2 DM, particularly those with multiple comorbidities or more severe respiratory involvement.

Future studies with larger sample sizes, longitudinal follow-up, and more comprehensive multivariable analyses are needed to clarify the independent effects of physical inactivity, obesity, disease duration, glycemic control, and comorbidities on respiratory muscle strength and cough performance in individuals with Type 2 DM.

## 5. Conclusions

In conclusion, individuals with Type 2 DM demonstrated lower physical activity levels, respiratory muscle strength, and cough strength than healthy controls. Higher physical activity levels were associated with better respiratory muscle strength and cough strength in individuals with Type 2 DM. In addition, longer diabetes duration was associated with lower inspiratory muscle strength. Although cough strength values in the Type 2 DM group remained above the threshold generally considered necessary for effective cough function, they were significantly lower than those in healthy controls. Furthermore, the associations between Type 2 DM and both inspiratory muscle strength and cough strength remained significant after adjustment for BMI and waist circumference, suggesting that these impairments cannot be explained solely by obesity-related factors. These findings indicate that respiratory muscle function and cough performance may be adversely affected in individuals with Type 2 DM and warrant further investigation in larger longitudinal studies.

## Figures and Tables

**Table 1:** Demographic characteristics and laboratory findings of type 2 DM and healthy control groups.

Variable	Type 2 DM Group (Median, Q1–Q3)	Healthy Control Group (Median, Q1–Q3)	z	*p*
Age (years)	52.0 (43.5–62.75)	52.0 (43.5–62.75)	0.000	1
Weight (kg)	85.5 (73.25–95.0)	74.5 (62.25–87.0)	−2.026	0.043 *
Height (cm)	160.0 (154.5–164.75	165.0 (160.0–171.75)	−1.681	0.093
BMI (kg/m^2^)	32.23 (28.25–37.73)	28.29 (24.42–30.25)	−2.794	0.005 *
Waist (cm)	125.0 (101.25–139.75)	92.0 (85.0–107.0)	−4.478	0.001 *
Hip (cm)	110.0 (101.0–115.5)	103.0 (97.0–108.25)	−2.343	0.019 *
Waist/Hip	1.11 (1.01–1.25)	0.87 (0.85–1.03)	−4.606	0.001 *
SBP (mmHg)	125.0 (120.0–133.75)	120.0 (120.0–125.0)	−2.310	0.021 *
DBP (mmHg)	80.0 (80.0–80.0)	75.0 (70.0–80.0)	−3.951	0.001 *
Fasting blood glucose (mg/dL)	153.5 (100.0–204.0)	91.0 (86.25–97.0)	−4.498	0.001 *
Post-prandial blood glucose (mg/dL)	222.0 (162.07–369.0)	115.5 (105.25–122.5)	−5.945	0.001 *
HDL (mg/dL)	50.5 (41.0–73.5)	52.4 (45.25–60.0)	−0.148	0.882
LDL (mg/dL)	99.0 (85.75–116.0)	125.0 (103.75–142.0)	−2.736	0.006 *
Total cholesterol (mg/dL)	180.5 (162.75–204.75)	188.0 (173.0–199.0)	−0.348	0.728
Triglyceride (mg/dL)	145.5 (93.5–246.75)	98.5 (84.25–118.5)	−2.676	0.007 *
Vitamin B12 (pg/mL)	330.3 (249.75–428.85)	363.0 (280.25–499.25)	−0.695	0.487
HbA1c (%)	8.35 (7.12–11.05)	5.20 (4.83–5.50)	−6.662	0.001 *
Folic acid (ng/mL)	8.22 (7.34–10.07)	7.96 (6.14–9.66)	−1.153	0.249

BMI: body mass index; SBP: systolic blood pressure; DBP: diastolic blood pressure; HDL: high-density lipoprotein; LDL: low-density lipoprotein; HbA1c: hemoglobin A1c. Values are reported as median (25th–75th percentile). z: Mann–Whitney U test; * *p* < 0.05.

**Table 2:** Cough strength, respiratory muscle strength, and physical activity levels in the type 2 DM and healthy control groups.

Variable	Type 2 DM Group (Median, Q1–Q3)	Healthy Control Group (Median, Q1–Q3)	z	*p*
Cough Strength (L/min)	250.0 (200.0–357.5)	400.0 (340.0–450.0)	−4.081	0.001 *
MIP (cmH_2_O)	55.0 (42.5–69.5)	70.5 (66.25–80.75)	−3.853	0.001 *
MEP (cmH_2_O)	62.0 (48.5–69.25)	79.0 (73.25–86.75)	−4.526	0.001 *
MIP %	78.0 (59.0–85.75)	101.5 (88.25–113.75)	−3.786	0.001 *
MEP %	59.0 (50.0–73.5)	87.5 (76.25–100.0)	−3.845	0.001 *
IPAQ (MET min/week)	696.5 (340.88–1073.25)	1530.0 (714.0–2189.25)	−2.632	0.008 *

MIP: maximal inspiratory pressure; MEP: maximal expiratory pressure; IPAQ: International Physical Activity Questionnaire. Values are reported as median (25th–75th percentile). z: Mann–Whitney U test; * *p* < 0.05.

**Table 3:** Relationship between IPAQ score, cough strength, respiratory muscle strength, and HbA1c levels in the Type 2 DM and healthy control groups.

Variable	IPAQ (MET Min/Week)			
	Type 2 DM Group		Healthy Control Group	
	r	*p*	r	*p*
Cough Strength (L/min)	0.619	<0.001 *	0.470	0.010 *
MIP (cmH_2_O)	0.438	0.016 *	0.264	0.166
MEP (cmH_2_O)	0.581	0.001 *	0.466	0.011 *
HbA1c (%)	−0.352	0.056	0.018	0.928

MIP: maximal inspiratory pressure; MEP: maximal expiratory pressure; IPAQ: International Physical Activity Questionnaire; HbA1c: hemoglobin A1c. * *p* < 0.05.

**Table 4:** Relationship between clinical characteristics, physical activity level, and respiratory outcomes in the Type 2 DM group.

Variable	Diabetes Duration		BMI		HbA1c (%)		WHR	
	r	*p*	r	*p*	r	*p*	r	*p*
Cough Strength (L/min)	−0.357	0.053	−0.108	0.570	−0.352	0.056	−0.106	0.578
MIP (cmH_2_O)	−0.412	0.024 *	−0.058	0.760	−0.369	0.045 *	−0.047	0.758
MEP (cmH_2_O)	−0.261	0.164	−0.291	0.119	−0.102	0.593	−0.148	0.822
IPAQ (MET min/week)	−0.029	0.878	−0.095	0.616	−0.352	0.056	−0.246	0.190

BMI: body mass index; HbA1c: hemoglobin A1c; MIP: maximal inspiratory pressure; MEP: maximal expiratory pressure; IPAQ: International Physical Activity Questionnaire. Correlations were analyzed using Spearman’s correlation coefficient. * *p* < 0.05.

**Table 5:** Multivariable regression analyses examining the association between group status and respiratory outcomes after adjustment for BMI and waist circumference.

Dependent Variable	B (Group: Type 2 DM vs. Healthy Controls)	95% CI	*p* Value
MIP (cmH_2_O)	−15.69	−26.58 to −4.80	0.006 *
MEP (cmH_2_O)	−14.29	−29.05 to 0.46	0.057
Cough Strength (PEF, L/min)	−90.51	−161.52 to −19.50	0.013 *

Models were adjusted for BMI and waist circumference. Β represents the unstandardized regression coefficient for group status. MIP: maximal inspiratory pressure; MEP: maximal expiratory pressure; PEF: peak expiratory flow; CI: confidence interval. * *p* < 0.05.

## Data Availability

The data presented in this study are available on reasonable request from the corresponding author.

## References

[1] Sharma J.K., Gupta A., Khanna P. (2019). Diabetes and respiratory system including tuberculosis–challenges. Indian J. Tuberc..

[2] International Diabetes Federation (2025). IDF Diabetes Atlas.

[3] Pongpanit K., Kulchanarat C., Buranapuntalug S. (2019). Correlation between change in respiratory muscle strength and cough ability in patients submitted to open-heart surgery. East. J. Med..

[4] Tosta A.M., Borges M.C., Silva É.M.C., Silva A.A., Crema E. (2022). Pre- and postoperative respiratory muscle strength, body mass index and fasting glucose profile of patients with type 2 diabetes mellitus submitted to metabolic surgery. Fisioter. Mov..

[5] Van den Borst H.R., Gosker M.P., Zeegers A. (2010). Pulmonary function in diabetes: A meta-analysis. Chest.

[6] Carvalho T.D., Pastre C.M., de Godoy M.F. (2011). Fractal correlation property of heart rate variability in chronic obstructive pulmonary disease. Int. J. Chronic Obstr. Pulm. Dis..

[7] Patel N., Howard I.M., Baydur A. (2023). Respiratory considerations in patients with neuromuscular disorders. Muscle Nerve.

[8] Brennan M., McDonnell M.J., Duignan N., Gargoum F., Rutherford R.M. (2022). The use of peak cough flow in the assessment of respiratory function in clinical practice: A narrative review. Respir. Med..

[9] Al-Khlaiwi T., Alsabih A.O., Khan A., Habib S., Sultan M., Habib S.S. (2021). Reduced pulmonary functions and respiratory muscle strength in type 2 diabetes mellitus and its association with glycemic control. Eur. Rev. Med. Pharmacol. Sci..

[10] Salam A., Tilluckdharry L., Amoateng-Adjepong Y., Manthous C.A. (2004). Neurologic status, cough, secretions and extubation outcomes. Intensive Care Med..

[11] Duan J., Zhang X., Song J. (2021). Predictive power of extubation failure diagnosed by cough strength: A systematic review and meta-analysis. Crit. Care.

[12] Meuffels F.M., Lichtmess C., Kreutz T., Held S., Brinkmann C. (2025). Self-Reported Physical Activity Among Individuals with Diabetes Mellitus—Identifying Potential Barriers and Facilitators. Diabetology.

[13] Biernat K., Marciniak D.M., Mazurek J., Kuciel N., Hap K., Kisiel M., Sutkowska E. (2024). The Level and Limitations of Physical Activity in Elderly Patients with Diabetes. J. Clin. Med..

[14] Vasconcelos A.R., Guimarães F.J.S.P., Cruz P.W.S., Carvalho M.J.M.C.B., Brito A.F., Costa K.B., Figueiredo L.S., Schwingel P.A., Vancea D.M.M., Costa M.C. (2025). Sarcopenia and Functional Decline in Postmenopausal Women: The Roles of Type 2 Diabetes and Physical Activity. Med. Sci..

[15] Persiani M., Laffi A., Piras A., Meoni A., Brodosi L., Nicastri A., Petroni M.L., Raffi M. (2025). Relationship Between Physical Activity and Autonomic Responses in Adults with Type 2 Diabetes. Int. J. Environ. Res. Public Health.

[16] Lecky R., Grogan S., Shukla P., Atkinson S., McClean P.L., Kelly C. (2026). The Role of Obesity and Type 2 Diabetes in Lung Health: A Systematic Review. PLoS ONE.

[17] Breuil-Marsal Z., Godek C., Lotti A., Feiereisen P., Marçal I.R., Rehder-Santos P., Milan-Mattos J.C., de Abreu R.M. (2024). Acute and Chronic Effects of Inspiratory Muscle Training in Patients with Type 2 Diabetes Mellitus: A Systematic Review of Randomized Controlled Trials. Front. Sports Act. Living.

[18] American Diabetes Association (2024). Classification and diagnosis of diabetes: Standards of Care in Diabetes—2024. Diabetes Care.

[19] Fuso L., Pitocco D., Longobardi A. (2012). Reduced respiratory muscle strength and endurance in type 2 diabetes mellitus. Diabetes Metab. Res. Rev..

[20] Simões R.P., Deus A.P., Auad M.A., Dionísio J., Mazzonetto M., Borghi-Silva A. (2010). Maximal respiratory pressure in healthy 20 to 89 year-old sedentary individuals of central São Paulo State. Braz. J. Phys. Ther..

[21] Takeda H., Yamashina Y., Tabira K. (2022). Relationship between kyphosis and cough strength and respiratory function of community-dwelling elderly. Physiother. Theory Pract..

[22] Nunn A.J., Gregg I. (1989). New regression equations for predicting peak expiratory flow in adults. BMJ.

[23] Sibai A.M., Costanian C., Tohme R. (2013). Physical activity in adults with and without diabetes: From the high-risk approach to the population-based approach of prevention. BMC Public Health.

[24] Köklü N., Büyüköztürk Ş., Bökeoğlu Ç.Ö. (2006). Sosyal Bilimler İçin İstatistik.

[25] Freitas F.S., Ibiapina C.C., Alvim C.G., Britto R.R., Parreira V.F. (2010). Relationship between cough strength and functional level in a group of elderly individuals. Braz. J. Phys. Ther..

[26] Razak S., Justine M. (2020). Relationship between respiratory muscle functions, obesity indices, and body compositions at different levels of physical activity in young adults. J. Exerc. Physiol. Online.

[27] Silva A., Silva L., Lopes I., Francisco A., Neto A., Monteiro M., Muela H. (2024). Association of dietary pattern and physical inactivity with hypertension, obesity, diabetes and metabolic syndrome. Metabolic Syndrome–Lifestyle and Biological Risk Factors.

[28] Saxena Y., Sidhwani G., Upmanyu R. (2009). Abdominal obesity and pulmonary functions in young Indian adults: A prospective study. Indian J. Physiol. Pharmacol..

[29] Bach J.R., Gonçalves M.R., Páez S., Winck J.C., Leitão S., Abreu P. (2006). Expiratory flow maneuvers in patients with neuromuscular diseases. Am. J. Phys. Med. Rehabil..

[30] Suárez A.A., Pessolano F.A., Monteiro S.G., Ferreyra G., Capria M.E., Mesa L., Dubrovsky A., De Vito E.L. (2002). Peak flow and peak cough flow in the evaluation of expiratory muscle weakness and bulbar impairment in patients with neuromuscular disease. Am. J. Phys. Med. Rehabil..

[31] Kim J., Davenport P., Sapienza C. (2009). Effect of expiratory muscle strength training on elderly cough function. Arch. Gerontol. Geriatr..

[32] Winck J.C., LeBlanc C., Soto J.L., Plano F. (2015). The value of cough peak flow measurements in the assessment of extubation or decannulation readiness. Rev. Port. Pneumol. (Engl. Ed.).

[33] Al-Rifai R.H., Pearson F., Critchley J.A. (2017). Association between diabetes mellitus and active tuberculosis: A systematic review and meta-analysis. PLoS ONE.

